# Evaluate the Correlation between the TIMI Frame Count, IMR, and CFR in Coronary Microvascular Disease

**DOI:** 10.1155/2022/6361398

**Published:** 2022-02-02

**Authors:** Xinye Xu, Jinbao Zhou, Yongzhen Zhang, Qian Li, Lijun Guo, Yanyang Mao, Liyun He

**Affiliations:** ^1^Department of Cardiology and Institute of Vascular Medicine, Peking University Third Hospital, NHC Key Laboratory of Cardiovascular Molecular Biology and Regulatory Peptides, Key Laboratory of Molecular Cardiovascular Science, Ministry of Education, Beijing Key Laboratory of Cardiovascular Receptors Research, Beijing, China; ^2^Department of Cardiovascular Disease, Peking University Third Hospital, Yan'an Branch, Yan'an, China

## Abstract

**Objective:**

To evaluate the correlation between the TIMI frame count, IMR, and CFR in coronary microvascular disease (slow flow phenomenon).

**Methods:**

TFC and IMR were recorded in the nitroglycerin and ATP administration states, and the relationship between TFC, IMR, and CFR in specific states was analyzed.

**Results:**

A total of 41 patients with baseline TFC >25 frames on coronary angiography were enrolled, and nitroglycerin reduced TFC by 50% from baseline in 24 (58.54%) patients; 16 of the remaining 17 patients were able to achieve a 50% reduction in TFC by further intracoronary ATP injection. 10 patients were further tested for IMR, and the results showed significant correlations between baseline TFC and IMR (*r* = 0.775, *P*=0.008), TFC and IMR after nitroglycerin (*r* = 0.875, *P*=0.001), and the minimal TFC and IMR that could be obtained with nitroglycerin or ATP administration (*r* = 0.890, *P*=0.001). There was also a significant correlation between the proportional improvement in TFC and CFR before and after nitroglycerin injection (*r* = 0.685, *P*=0.029). In addition, we observed a lower IMR measured after nitroglycerin than after ATP in three patients, suggesting that CMD may be dominated by NO-sensitive vascular such as prearterioles and that an extensive analysis of the target site of CMD may be achieved by stepwise drug administration.

**Conclusion:**

Induction of TFC in different states by a stepwise drug approach may serve as a potential primary screening method for coronary microcirculatory dysfunction, thereby reducing the need for further IMR or CFR testing.

## 1. Introduction

Coronary microvascular dysfunction (CMD) is a clinical phenomenon that causes myocardial ischemia due to functional or structural abnormalities of the microvascular and is considered one of the causes of nonobstructive coronary heart disease [1]. The invasive diagnostic criteria for CMD mainly include coronary slow flow (CSF) phenomenon presenting as TIMI frame count (TFC) >25 frames, index of microcirculatory resistance (IMR) >25, or coronary flow reserve (CFR) <2.0 [2]. Some studies have shown that there is no significant correlation between the three indices, suggesting that these three indices are measured from different dimensions [3, 4]. TFC evaluates the state of CMD at the baseline status; IMR evaluates the state when the microcirculation is fully dilated, focusing more on structural CMD; and CFR reflects the reserve capacity of coronary microcirculation. From this, we can also see that the difference between the three is related to different states at the time of testing, in addition to different testing methods. Therefore, we envisage that it is possible to find a connection between the three and even to substitute each other to some extent if the tests are performed in the same state. Due to the huge differences in the complexity of the detection methods, it is worth discussing whether TFC, a relatively easy-to-implement detection method, can replace the complex detection methods of IMR and CFR after its process improvement to achieve the goal of simplifying the CMD assessment process.

## 2. Method

### 2.1. Patient Enrollment

Patients with coronary angiography showing slow flow were enrolled consequently. CSF was defined as the TIMI frame count >25 frames at 15 frames per second (fps). The clinical diagnosis in all patients favored microvascular angina, i.e., coronary angiography showed no >50% stenosis or significant thrombotic, dissection lesions in the large epicardial vessels; also, there was no evidence of typical variant angina-epicardial large vessel spasm.

### 2.2. Coronary Angiography, Intracoronary Drug Administration, and TIMI Frame Count

Coronary angiography was performed using a 5 F TIG catheter through the radial artery. The contrast medium was injected manually by the same operator, and it was required to ensure satisfactory filling of the intravascular contrast medium and clear display of the vessel contour. The image acquisition frequency was standard 15 frames per second (fps), with a blank phase, and the image acquisition was maintained until the last branch of the distal coronary artery was visualized [5]. LAD was selected for all patients. The TFC result is obtained by subtracting the number of frames of the opening from the most distal branch. Each patient will undergo 2-3 steps of TIMI counting. In the first step, baseline TIMI frame (TFC_Base_) will be recorded without any intracoronary medication; in the second step, TIMI frame (TFC_NIT_) will be recorded after intracoronary injection of 200 *μ*g of nitroglycerin; if the decrease in TFC_NIT_ compared to TFC_Base_ is less than 50%, then the third step is followed—inject 100 *μ*g of adenosine triphosphate intracoronarily and record the TFC once again (TFC_ATP_). It is important to note that there should be at least a 2-minute interval between the nitroglycerin and ATP injections and to confirm that the TFC returns to baseline levels to ensure complete elution of the nitroglycerin effect.

### 2.3. Measurement of IMR, CFR, and FFR

IMR, CFR, and fractional flow reserve (FFR) were evaluated in all patients who signed an informed consent form using a C12008 pressure wire (Abbott, Illinois, USA) following a standard procedure. Blood was flushed out of the system with saline prior to testing, and the pressure of the catheter system was equalized to the atmospheric pressure and the pressure of the catheter system before the pressure guide wire entered the catheter. The test was performed using a 6 F guide catheter, and the pressure guide wire was equalized to the system pressure when it reached the ostium of the LCA. The pressure guide wire was advanced as far as possible into the distal segment of the LAD while avoiding wall attachment or significant tension on the tip of the pressure guide wire.

After confirming that the guiding catheter and LCA ostium were well engaged, intracoronary bullet injections of 5 ml of room temperature saline were performed to obtain the mean transit time (T_mn_), and mean distal pressure (Pd) was recorded simultaneously [6, 7]. Pd and T_mn_ were acquired in 3 specific states: baseline state (without any vasoactive drug), after intracoronary injection with 200 ug nitroglycerin, and during continuous intravenous pumping of adenosine triphosphate (160 ug/kg ∗ min). After each specific state is tested, the next state is waited for the TIMI frame count to return to baseline levels.

IMR was then calculated as Pd × T_mn_. Base, NIT, and ATP were used as footnotes for the IMR in the corresponding states, which were presented as IMR_Base_, IMR_NIT_, and IMR_ATP_. The lowest IMR that can be achieved after NIT or ATP treatment was represented by IMR_Min_.

CFR was calculated as the ratio of T_mn_ at rest to T_mn_ at the maximum hyperemic state. Use the footer to indicate the comparison of the two states in which the CFR occurs. For example, a comparison between the baseline state and the nitroglycerin-induced state is indicated as CFR_Base/NIT_.

FFR was calculated as Pd/Pa in the maximal hyperemic state. The maximal hyperemic state was obtained by intracoronary nitroglycerin injection with continuous intravenous pumping of adenosine triphosphate (160 ug/kg ∗ min).

### 2.4. Statistical Methods

Statistical analyses were performed using SPSS 26.0 software (IBM Corp., Armonk, NY, USA). For continuous variables, the Kolmogorov–Smirnov test was used to determine whether the data are normally distributed. Continuous variables were presented as mean ± standard deviation or median (quartile), whereas gender, past medical history, etc., were presented as percentages. Spearman's correlation analysis was used to analyze the correlation between parameters. A two-tailed *P* value <0.05 was considered statistically significant.

## 3. Results

### 3.1. General Data

A total of 41 patients were enrolled, including 28 males and 13 females, with a mean age of 59.02 ± 12.01. All patients were admitted with chest pain/discomfort, but not exertional. 24 had a history of hypertension, 17 had type 2 diabetes mellitus, 21 patients (51.22%) were current smokers, and the proportion of other comorbid diseases was low. The mean BMI was 25.01 ± 3.72 kg/m^2^ in males and 24.92 ± 3.24 kg/m^2^ in females, both meeting the criteria for overweight. Mean systolic blood pressure was 125.39 ± 16.89 mmHg, diastolic blood pressure was 75.95 ± 10.83 mmHg, and heart rate was 72.05 ± 11.39 bpm. Ancillary examinations showed that mean HbA1c was 6.80 ± 1.50%, NT-proBNP was 77 (5, 1871) pg/ml, and mean LVEF was 63.95 ± 6.47%. Detailed examination results are shown in Table 1.

### 3.2. Result of TFC Analysis

**Figure 1:**
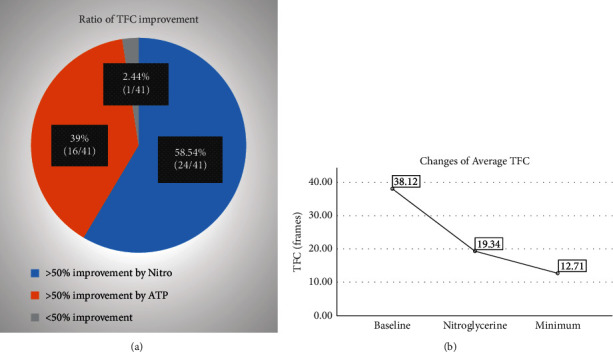
(a) The proportion of TFC improved by nitroglycerin or ATP; (b) the extent to which TFC can be reduced after intracoronary nitroglycerin injection and after sequential ATP injection.

Mean TFC_Base_ for all 41 patients was 38.12 ± 10.12 frames, and mean TFC_Nit_ was 19.34 ± 8.39 frames, with a mean reduction in TFC_Nit_ over TFC_Base_ of 49.40 ± 15.91%. The proportion of patients with >50% reduction was 58.54% (24/41), and their baseline median frames were 37 (29, 41) frames, which decreased to 14 (12, 17) frames with nitroglycerin, with a median reduction of 60.49% (54.90%, 64.39%); the proportion of patients with ≦50% reduction was 41.46% (16/41), and their baseline median frames were 36 (29, 49) frames, which decreased to 25 (18, 34) frames with nitroglycerin, with a median decrease of 36.11% (32.35%, 40.01%). There was no statistically significant difference between the two groups of effective/ineffective nitroglycerin in TFC_Base_ (*P*=0.519) (Figure 1).

For the 17 patients with <50% reduction in TFC_Base_ induced by nitroglycerin, TFC_ATP_ was assayed after nitroglycerin elution, and the results were 11 (9, 15) frames with a median reduction of 69.44% (65.48%, 72.43%) compared to TFC_Base_. All patients could obtain more than 50% reduction in the TFC from baseline by nitroglycerin or ATP injection, except for 1 patient who had a reduction of less than 50% from baseline.

### 3.3. Result of Specific IMR

**Figure 2:**
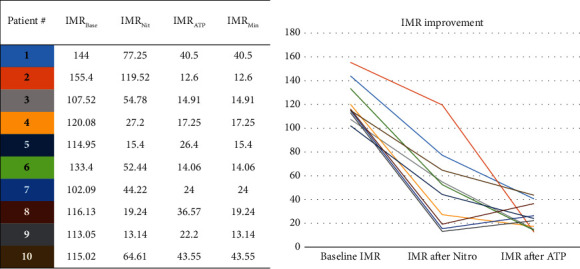
10 cases underwent IMR examination. The baseline IMR (IMR_Base_) was distributed between 100 and 160, and 3 patients had higher IMR induced by ATP (IMR_ATP_) than IMR induced by nitroglycerin (IMR_Nit_), suggesting difference in the position of functional microcirculatory insufficiency.

A total of 10 patients underwent examination with the pressure wire, and FFR was above 0.87 in all patients (0.87 to 0.96). Six of them had >50% reduction in the TFC induced by nitroglycerin, three had >50% reduction in the TFC induced by ATP, and one could not achieve >50% reduction in the TFC by both nitroglycerin and ATP. The results showed that the included patients had TFC_Base_ of 32 (28, 39) frames and TFC_NIT_ of 15 (12, 22) frames, and the minimum TFC induced by nitroglycerin or ATP (TFC_Min_) was 12 (10, 14) frames. IMR_Base_ was 115.58 (111.67, 136.05), IMR_NIT_ was 48.33 (18.28, 67.77), IMR_ATP_ was 23.10 (14.70, 37.55), and the minimum IMR induced by nitroglycerin or ATP (IMR_Min_) was 16.33 (13.83, 28.13). Three patients had IMR_Nit_ <25, six patients had IMR_ATP_ <25, and there was no correlation between IMR_Nit_ and IMR_ATP_ (Figure 2).

### 3.4. Correlation of TFC with Specific IMR

**Figure 3:**
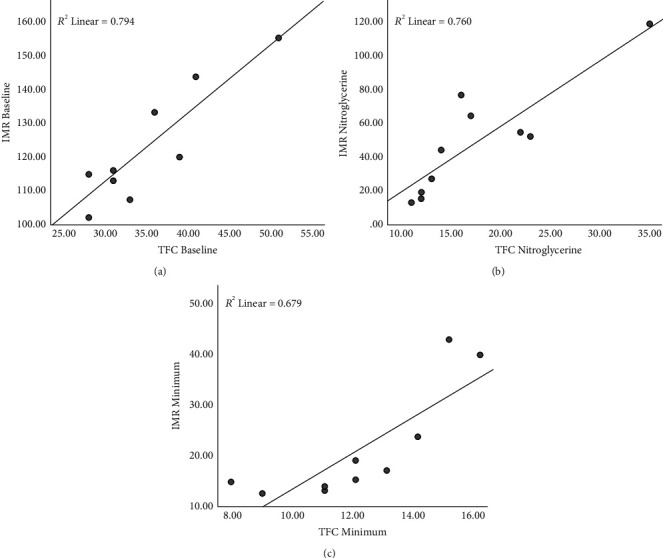
Correlation between IMR and TFC. (a) TFC_Base_ was significantly correlated with IMR_Base_ (*r* = 0.775, *P*=0.08). (b) TFC_Nit_ was significantly correlated with IMR_Nit_ (*r* = 0.875, *P*=0.001). (c) TFC_Min_ was significantly correlated with IMR_Min_ (*r* = 0.890, *P*=0.001).

After treatment with nitroglycerin or ATP, IMR_Min_ decreased to below 25 in 8 patients. The other 2 patients had IMR_Min_ of 40.50 and 43.55, respectively, and their corresponding TFC_Min_ was 16 and 15 frames, respectively. Spearman's correlation analysis showed that TFC_Base_ was significantly correlated with IMR_Base_ (*r* = 0.775, *P*=0.008), but not with IMR_NIT_, IMR_ATP_, or IMRMin. TFC_Nit_ was significantly correlated with IMR_Nit_ (*r* = 0.875, *P*=0.001), and TFC_Min_ was significantly correlated with IMR_Min_ (*r* = 0.890, *P*=0.001) (Figure 3).

### 3.5. Correlation of TFC and CFR

**Figure 4:**
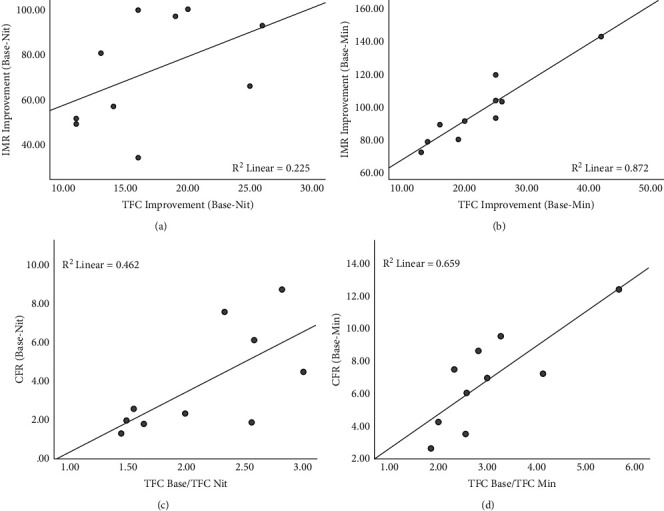
Correlation between the improvement of TFC and IMR. (a) No significant correlation in the actual value improvement between TFC_Base-Nit_ and IMR_Base-Nit_ (*r* = 0.549, *P*=0.10). (b) A significant correlation between the actual value improvement between TFC_Base-Min_ and IMR_Base-Min_ (*r* = 0.926, *P* < 0.001). (c, d) A significant correlation between the ratio of TFC_Base/Nit_ and CFR_Base/Nit_ (*r* = 0.685, *P*=0.029) as well as between TFC_Base/Min_ and CFR_Base/Min_ (*r* = 0.758, *P*=0.011). ^*∗*^Base: baseline; Nit: measured after nitroglycerin injection; ATP: measured after ATP injection; Min: measured after sequenced nitroglycerin and ATP injection.

The value of the decrease in TFC_Nit_ compared to TFC_Base_ was not significantly correlated with the value of the decrease in IMR_Nit_ compared to IMR_Base_ (*r* = 0.549, *P*=0.100) (Figure 4(a)), while the value of the decrease in TFC_Min_ compared to TFC_Base_ was significantly correlated with the value of the decrease in IMR_Nit_ compared to IMR_Base_ (*r* = 0.926, *P* < 0.001) (Figure 4(b)).

We calculated the ratio of TFC (TFC_Base/Nit_) and CFR (CFR_Base/Nit_) for the coronary nitroglycerin-injected state compared with the baseline state and the maximum ratio of TFC (TFC_Base/Min_) and CFR (CFR_Base/Min_) that could be obtained for the nitroglycerin-injected or ATP state compared with the baseline state. The results showed median TFC_Base/Nit_ of 2.17 (1.55, 2.54) and median CFR_Base/Nit_ of 2.43 (1.84, 6.39), showing a significant correlation (*r* = 0.685, *P*=0.029) (Figure 4(c)). Median TFC_Base/Min_ was 2.70 (2.25, 3.49), and median CFR_Base/Min_ was 7.09 (4.08, 8.82), and there was a significant correlation between them (*r* = 0.758, *P*=0.011) (Figure 4(d)).

## 4. Discussion

CMD includes both functional and structural types, and some patients have both of these types, which are called mixed types. The incidence of structural abnormality is relatively low and is commonly caused by various causes of myocardial hypertrophy and atherosclerosis; functional CMD is relatively common and mainly results from vasospasm caused by various abnormalities in vasodilation and contraction regulation, which can usually be reversed using vasodilator drugs. Although there are differences in the technical principles of TFC, IMR, and CFR, what is more important is the difference in patient status at the time of testing. TFC is the state of CMD at baseline without medication and may contain both functional and structural factors, IMR is the state of maximal hyperemia induced by medication and retains only structural factors, whereas CFR evaluates the effect of medication and corresponds to the weighting of functional factors. Therefore, it is not reasonable to directly compare the three parameters and obtain results without correlation in previous studies. A more reasonable approach would be to compare the clinical values of the three parameters in the same hyperemic state.

In the present study, TFC and IMR were recorded separately for baseline and drug-induced maximum hyperemic states. Under the same state, TFC and IMR showed a good correlation, and there was also a significant correlation between improvement in TFC and CFR. Since TFC is a simpler method than CFR and IMR, the above results suggest a possible initial screening of coronary microcirculatory function by the results of TFC in different states to exclude the majority of patients with functional CMD. Only patients with insufficient drug effect require further IMR. To achieve this, there are still some issues that need further work to be addressed.

To further ensure the consistency of TFC and IMR in terms of measurement status, the choice of the target artery to be measured may have to be changed. The LAD was used in this study, which may not be a good choice. Since TFC is usually measured using an angiographic catheter, while IMR requires a guiding catheter, and the morphology of the LCA ostium and the diameter of the LM are highly variable, there are differences in the engagement of the LCA ostium between the two catheters, further affecting the coaxiality and filling speed of the contrast in the LAD. RCA, on the contrary, has relatively few problems in this regard and may be a more appropriate choice. Of course, this choice can only be made if it is not a significant left dominant, and RCA also shows a significant slow flow. The use of an extension catheter for the LAD with larger internal diameters may also be an option. This problem mainly affects the stability and reproducibility of the assay and is also present in the IMR measurement process.

In-depth consideration is also needed in the selection of vasodilator drugs to achieve maximum hyperemia. The coronary microvasculature is a complex network that includes three components: prearterioles (200–400 um), arterioles (20–200 um), and capillaries. Both prearterioles and arterioles are the main regulatory vessels and can be classified as nitric oxide-dependent and nondependent, with differences in response to drugs. Adenosine/ATP acts mainly in 20–200 um vessels [8], whereas vessels in the 200–400 um diameter range are more sensitive to nitrates [9]. Depending on the vascular response to the drug, it can also be classified as endothelium-dependent dysfunction, nonendothelium-dependent dysfunction, or a combination of the two types. Of course, this functional classification is also related to the anatomical segment [10]. Although the classic approach to diagnosing CMD is to use adenosine/ATP alone, acetylcholine can identify endothelium-dependent dysfunction and increase diagnostic accuracy. However, there are risks associated with the process of acetylcholine-induced microcirculatory spasm [11, 12].

In this study, we used a stepwise administration strategy: (1) nitroglycerin was used instead of acetylcholine in patients with CSF to determine the presence of endothelium-dependent dysfunction; (2) nitroglycerin was first given to observe the effect of nitroglycerin on CSF, and ATP was given to patients who responded poorly to nitroglycerin. We observed a “biphasic” improvement in TFC, with nitroglycerin, which could reduce TFC by more than 50% in 58.54% of patients, suggesting that the CSF in this group of patients was mainly from the spasm of the prearterioles; and the phenomenon that ATP was effective suggests that the site of their coronary spasm may be located in the arterioles. The phenomenon of lower IMR_NIT_ than IMR_ATP_ observed in some patients further suggests variability in the site of spasticity, the exact mechanism and clinical implications of which need to be further investigated. Thus, the stepwise administration strategy of nitroglycerin and ATP can be used to identify endothelium-dependent or nonendothelium-dependent, while a combination strategy of nitroglycerin and ATP/adenosine or the use of drugs such as nicorandil/fasudil that have a dilating effect covering the entire microvascular network may be relevant in determining the presence of structural dysfunction [13, 14]. Whether the use of stepwise administration allows for further refinement in locating the target vessels responsible for functional dysfunction and its clinical value requires further study.

### 4.1. Limitation

This study is only a preliminary exploratory study with a small sample size, which does not provide sufficient evidence for the generalizability of the results and is insufficient to analyze the factors associated with different phenotypes, and larger clinical studies are currently underway. In the follow-up study, the study process needs to be further improved, and it is proposed to perform TFC testing in both nitroglycerin and ATP states in all patients to further determine the proportion of different sites of microcirculatory abnormalities in the population and to analyze the associated factors. The clinical significance of the variability in response to drugs needs to be further explored.

## 5. Conclusion

CMD is a highly prevalent phenomenon, and the current diagnostic criteria for TFC, CFR, and IMR have significant overlap in terms of underlying theory. The preliminary results of the present study give a first insight into the association between the three parameters. Further clarification of this relationship through a larger study would be valuable to optimize the overall process of coronary microcirculation assessment and the diagnosis of CMD.

## Figures and Tables

**Table 1:** General data of patients enrolled.

Indicator	Value
Age (y)	59.02 ± 12.01
Gender (female, %)	31.71 (13/41)
BMI male (kg/m^2^)	25.01 ± 3.72
BMI female (kg/m^2^)	24.92 ± 3.24
HR (bpm)	72.05 ± 11.39
SBP (mmHg)	125.39 ± 16.89
DBP (mmHg)	75.95 ± 10.83
NT-proBNP (pg/ml)	77 (5, 1871)
HbA1c (%)	6.80 ± 1.50
LDL-c (mmol/L)	2.04 ± 0.58
Triglyceride (mmol/L)	1.50 (1.00, 2.19)
eGFR (ml/min ∗ kg)	94.92 ± 12.64
LVEF (%)	63.95 ± 6.47
IVS (mm)	9.23 ± 1.37
LVPW (mm)	8.90 ± 1.10
LVEDd (mm)	45.66 ± 4.62
Smoker (%)	51.22 (21/41)
Hypertension (%)	58.54 (24/41)
Type 2 diabetes (%)	41.46 (17/41)
Obstructive sleep apnea syndrome (%)	4.88 (2/41)

## Data Availability

All the data generated or analyzed during this study are included in this published article.
